# Evaluation of alveolar ridge preservation using collagen and platelet-rich fibrin: A systematic review

**DOI:** 10.4317/jced.63789

**Published:** 2026-02-26

**Authors:** Julia Gil-Morales, Cosimo Galletti, Javier Flores-Fraile, Alice Rose Greethurst, Fulvia Galletti, Francisco Real-Voltas

**Affiliations:** 1 School of Dentistry, Department of Integrated Dentistry, International University of Catalonia, Sant Cugat del Vallès, Barcelona, Spain; 2 Kore University of Enna, Faculty of Medicine and Surgery, 94100, Enna, Italy; 3 Department of Surgery. University of Salamanca. Campus Miguel de Unamuno 37007, Salamanca. Spain; 4 Department of Biomedical and Dental Science and Morphological and Functional Imaging, University of Messina, 98166 Messina, Italy

## Abstract

**Background:**

Alveolar ridge resorption following tooth extraction may compromise future implant placement and aesthetic outcomes. Several biomaterials have been proposed to limit post-extraction dimensional changes, among which collagen-based materials and platelet-rich fibrin (PRF) are widely used. The aim of this systematic review was to evaluate the effectiveness of collagen and PRF in alveolar ridge preservation compared with spontaneous healing.

**Material and Methods:**

This systematic review was conducted according to PRISMA 2020 guidelines and registered in PROSPERO (CRD42024489237). An electronic search was performed in PubMed, Scopus to identify randomized controlled trials (RCTs) published from 2014 onwards. Studies conducted in adult humans that evaluated horizontal and/or vertical alveolar ridge changes after tooth extraction were included. Risk of bias was assessed using the Cochrane RoB 2 tool.

**Results:**

Sixteen RCTs were included. Collagen-based interventions, particularly when combined with bone grafts such as deproteinized bovine bone mineral (DBBM), demonstrated a significant reduction in vertical and horizontal bone loss compared with spontaneous healing in several studies. PRF-based protocols showed heterogeneous results regarding dimensional bone preservation, with limited benefit when PRF was used alone. However, PRF-especially advanced or recurrent applications-was consistently associated with improved soft tissue healing and reduced postoperative pain. Considerable heterogeneity was observed in biomaterial preparation, application protocols, follow-up duration, and outcome assessment.

**Conclusions:**

Both collagen and PRF can contribute to alveolar ridge preservation after tooth extraction, although their clinical benefits differ. Collagen, particularly when combined with bone grafts, appears more effective in preserving alveolar dimensions, while PRF provides greater advantages in soft tissue healing and postoperative pain control. The choice of biomaterial should be guided by the clinical objective and extraction site. Further well-designed, standardized long-term RCTs are needed to establish definitive clinical recommendations.

## Introduction

Dental extraction is one of the most frequently performed procedures in dentistry and can negatively impact the patient's quality of life, including aesthetic, functional, and psychological consequences (1 , 2). Common indications include extensive caries, non-restorable fractures, periodontal disease, orthodontic needs, failed endodontic or restorative treatments, periapical lesions, trauma, pathological lesions, or even patient request (3 , 4). A conservative extraction technique is essential to minimize alveolar trauma, reduce postoperative pain, and promote optimal healing. This involves the use of microsurgical instruments and careful detachment of the periodontal ligament to preserve the alveolar bone (1). Following tooth extraction, a biological healing cascade begins, leading to morphological changes in hard and soft tissues. Initially, a blood clot forms, which is gradually replaced by granulation tissue and subsequently new bone. This process can take up to 100 days to reach acceptable radiographic density (3). However, even when healing proceeds without complications, significant alveolar bone loss is common (5), especially during the first three months post-extraction, with estimated horizontal loss ranging from 20% to 63%, and vertical loss from 11% to 22% (6). Studies have shown that buccal bone resorption is more severe than lingual/palatal bone loss, resulting in a shift of the alveolar crest and creating anatomical limitations for implant placement and prosthetic rehabilitation (7 , 8). Most of this loss occurs within the first 90 days following extraction (9). As a result, several techniques have been developed to minimize post-extraction bone loss, including partial extraction therapies, orthodontic extrusion, and alveolar ridge preservation (ARP) (10). ARP, first documented in the 1960s, refers to surgical techniques applied immediately after tooth extraction to reduce external resorption and promote bone formation within the socket (3 , 11). While immediate implant placement may help preserve alveolar volume, it is not indicated in all cases, making ARP a valuable alternative (12). Common ARP procedures involve socket grafting with bone substitutes and sealing with collagen membranes or soft tissue grafts to prevent soft tissue ingrowth and facilitate guided tissue healing (13). The choice of biomaterial influences inflammation, revascularization, and bone integration. Therefore, understanding their biological properties-osteoconduction (serving as a scaffold), osteoinduction (stimulating osteoblast differentiation), and osteogenesis (direct bone formation by progenitor cells)-is crucial for clinical decision-making (8 , 14). Recent advances in biotechnology have led to the development of regenerative therapies such as Platelet-Rich Fibrin (PRF), an autologous biomaterial rich in platelets and leukocytes embedded in a fibrin matrix. PRF exhibits angiogenic, immunological, and regenerative properties and releases growth factors over several weeks, enhancing healing and tissue regeneration (15 , 16). Simultaneously, collagen sponges have emerged as a practical alternative to autogenous soft tissue grafts, acting as a physical barrier that supports epithelialization without requiring donor tissue. However, their rapid degradation has raised concerns about their long-term effectiveness in alveolar preservation (11 , 17). Despite the variety of techniques and materials available, no clear consensus exists on which method provides the best clinical and radiographic outcomes. Therefore, this systematic review aims to compare the effectiveness of two post-extraction ridge preservation techniques-collagen and PRF-against spontaneous healing, providing evidence-based guidance to support clinical decision-making in optimizing long-term outcomes. Although several systematic reviews have evaluated alveolar ridge preservation techniques in general, most of them have analyzed heterogeneous biomaterials collectively, without specifically comparing collagen-based approaches and platelet concentrates as distinct therapeutic strategies. To date, limited evidence has directly contrasted the differential impact of collagen and PRF on hard tissue dimensional stability versus soft tissue healing outcomes. Therefore, this review seeks to provide a more focused comparative synthesis of these two commonly used biomaterial families in clinical practice.

## Material and Methods

1. Search Strategy and Focused Question The search strategy used in this systematic review was based on the Preferred Reporting Items for Systematic Reviews and Meta-Analyses (PRISMA) 2020, and was registered in PROSPERO under the number CRD42024489237. The clinical question was formulated according to the PICO model . The question of systematic review was "Does the use of collagen or PRF improve clinical and radiographic outcomes in alveolar preservation compared to spontaneous healing?, focusing on: P - Population: Patients who have undergone tooth extraction I - Intervention: - Dental extraction and alveolar preservation with collagen application -Dental extraction and alveolar preservation with PRF (Platelet-Rich Fibrin) application C - Comparison: Dental extraction without alveolar preservation O - Outcome: Clinical and radiographic effects on the socket 2. Search Strategy Articles published and in press in the English language were electronically searched by two independent reviewers (J.G., A.R.) until 29 November 2024, with no restrictions concerning dates of coverage and publication status, across MEDLINE/PubMed, Scopus by applying the following keywords combined by Boolean operators (AND, OR and NOT): - "Alveolar preservation" (AND) "Collagen matrix" - "Tooth extraction" (AND) "Platelet rich Fibrin" - "Tooth extraction" (OR) "extraction" (OR) "dental extraction" (OR) "Tooth socket" (AND) "Alveolar ridge preservation" (OR) "ridge preservation" (OR) "Socket filling" (OR) "socket graft" (OR) "alveolar ridge preservation" (AND) "randomized controlled trial". 3. Study Selection Title and abstract assessment were accomplished for all the records identified through the database search. Two examiners (J.G.M and A.R.G) individually selected the studies in accordance with the inclusion criteria. Discrepancies were resolved by consensus. Full text reading was performed for articles considered suitable for the present systematic review based on the following inclusion and exclusion criteria (Table 1): 4. Risk of Bias Assessment The risk of bias of the included randomized controlled trials was assessed using the revised Cochrane Risk of Bias tool for randomized trials (RoB 2).

Table 1

**Figure 1:**
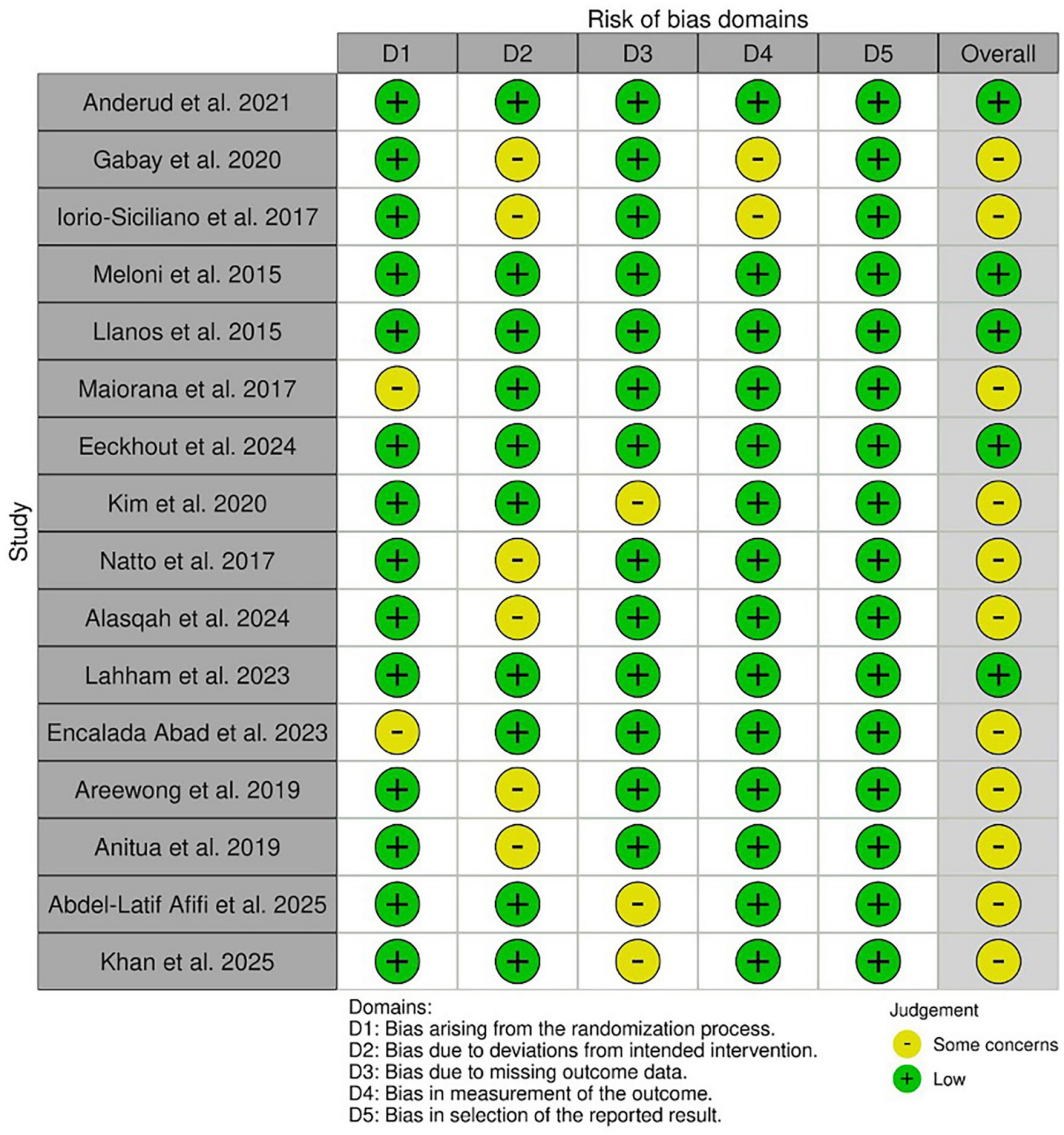
Risk of Bias assessment. According to the Cochrane RoB 2 tool.

Two reviewers independently evaluated each study, and any disagreements were resolved through discussion until consensus was reached. The following five domains were assessed: 1) bias arising from the randomization process, 2) bias due to deviations from the intended interventions, 3) bias due to missing outcome data, 4) bias in the measurement of the outcome, and 5) bias in the selection of the reported result. Each domain was judged as "low risk of bias," "some concerns," or "high risk of bias," according to the RoB 2 guidance. An overall risk of bias judgment was then assigned for each study. Trials were considered to be at low risk of bias when all domains were rated as low risk. Studies were classified as having some concerns if at least one domain raised some concerns but none were rated as high risk. An overall high risk of bias was assigned when at least one domain was judged as high risk. The results of the risk of bias assessment are summarized in Figure 1.

1

Due to substantial clinical and methodological heterogeneity among the included studies, a quantitative meta-analysis was not considered appropriate. The heterogeneity included variability in PRF preparation protocols (L-PRF, A-PRF+, C-PRF, PRGF), differences in collagen application (collagen alone versus collagen combined with bone grafts), variation in socket morphology (thin versus thick buccal wall), diverse outcome measures (millimeters of dimensional change versus percentage of new bone formation), and inconsistent follow-up periods ranging from 2 months to 12 months. These factors precluded meaningful statistical pooling of the data. Therefore, a structured qualitative synthesis was performed.

## Results

**Figure 2:**
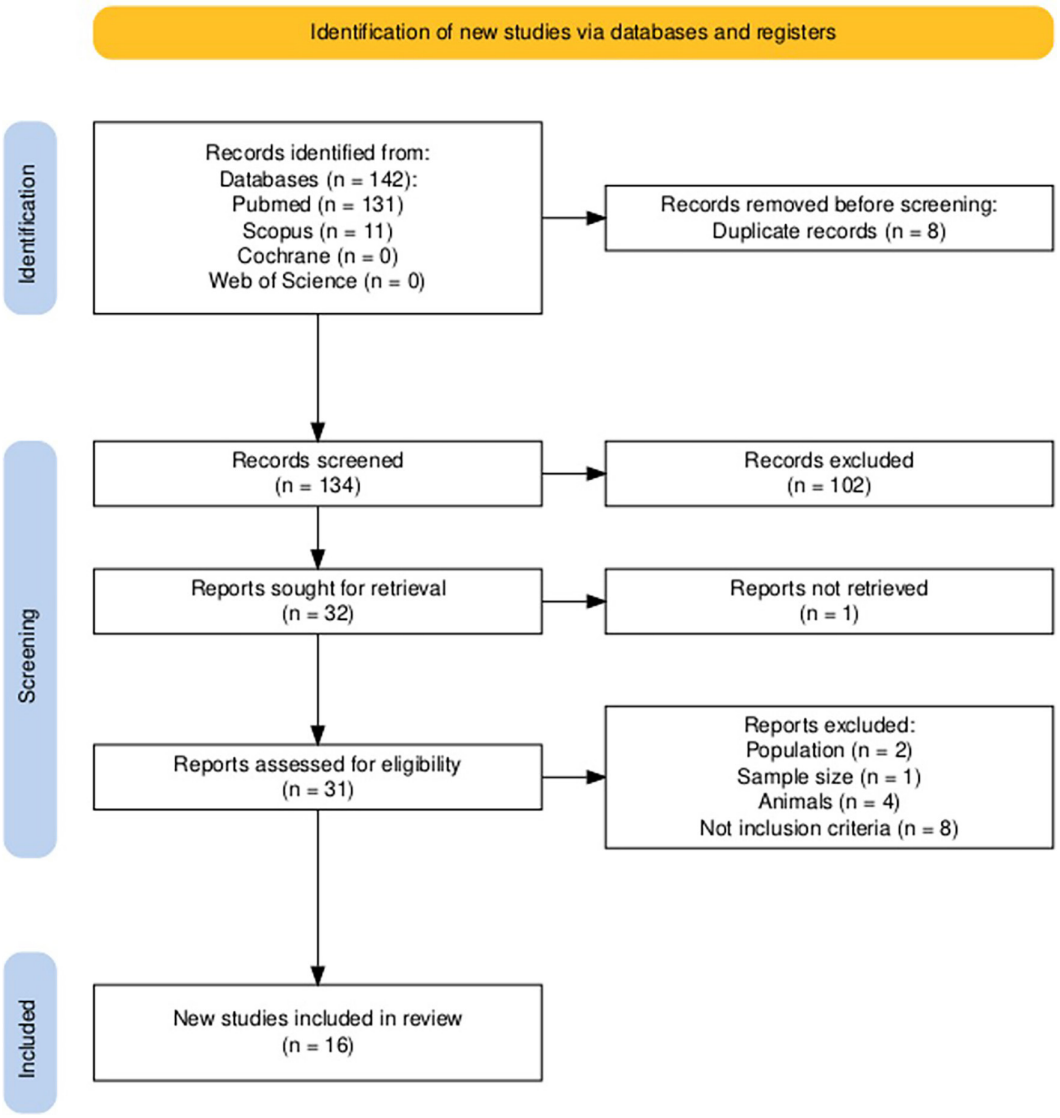
Flow Chart Diagram.

1. Study selection The study selection process illustrated in Figure 2, shows that after a widespread electronic search, 142 articles were identified: specifically, 131 from MEDLINE/PubMed and 11 from Scopus, as shown in the flow diagram created using the PRISMA Flow Diagram tool.

2

Following the removal of 8 duplicates, 134 were screened on the basis of titles and abstracts. Full text assessment was performed on 31 articles based on the inclusion criteria and 16 were finally selected for this present systematic review. All the present articles were RCTs. 3.2. Risk of Bias The risk of bias of the 16 randomized controlled trials was assessed using the Cochrane Risk of Bias Assessment Tool, version 2 (20). A total of 5 studies were classified as having a low risk of bias (n=5). The remaining studies (n=11) were classified as having some concerns, mainly due to issues related to Domain 2: bias due to deviations from the intended intervention. The results are presented in Figure 1.

3. Study Characteristics The clinical trials included in this systematic review focused on alveolar ridge preservation following tooth extraction in systemically healthy subjects over 18 years of age, although some authors set a higher age threshold. Eeckhout et al. chose to include patients over 21 years old (21), while Lahham et al. and Areewong et al. established a minimum inclusion age of 20 years (22 , 23). The duration of treatment varied considerably across studies. Anitua et al. and Anderud et al. analyzed their results 10-12 weeks after the intervention (24 , 25), Kim et al. followed their patients for 14 weeks (26), Areewong et al. reported a 2-month follow-up (23), and Alasqah et al. and Lahham et al. set a 3-month period (22 , 27). In longer-term studies, Llanos et al., Eeckhout et al., Natto et al., Abad et al., Maiorana et al., Afifi et al., and Khan et al. extended their observations up to 6 months (13 , 17 , 21 , 28 - 31). Meloni et al. maintained an even longer follow-up, extending up to one year post-extraction (32). The interventions used in the studies were diverse and included a variety of materials and approaches, based on the inclusion criteria of this systematic review. Some studies, such as those by Anderud et al., Eeckhout et al., and Kim et al., used collagen-based materials (21 , 25 , 26). Gabay et al., Iorio-Siciliano et al., Meloni et al., Llanos et al., and Maiorana et al. used bone substitutes, including deproteinized bovine bone mineral (DBBM) and xenografts (11 , 28 , 29 , 32 , 33). Other studies investigated the use of platelet-rich fibrin (PRF) and its derivatives, including those by Alasqah et al., Lahham et al., Abad et al., Areewong et al., Anitua et al., Afifi et al., and Khan et al. (13 , 22 - 24 , 27 , 30 , 31). Among the latter group, the procedures for obtaining plasma varied among authors. Alasqah et al. collected 9 to 10 mL of blood in tubes without anticoagulants, centrifuged at 2700 rpm for 12 minutes, generating a fibrin clot which was then compressed to form a PRF plug (27). Lahham et al. used a similar approach, drawing 20 mL of blood into two sterile tubes, centrifuged at 1300 rpm for 8 minutes, also obtaining fibrin clots that were compressed (22). Abad et al. described a more complex procedure, using eight 9 mL tubes centrifuged at 2700 rpm for 12 minutes, and manipulating the resulting clots to obtain both fibrin plugs and membranes (13). Areewong et al. described a method in which 10 mL of blood was drawn from superficial veins, and after centrifugation at 2700 rpm for 12 minutes, the fibrin clot was shaped into a plug using a compression box (23). Anitua et al. drew 36 mL of blood into tubes containing sodium citrate as an anticoagulant, centrifuging the sample at 580×g for 8 minutes. The plasma was then fractionated to obtain fibrin, which was activated before surgery to generate clots and membranes (24). Afifi et al. followed a process similar to other studies, collecting 5 mL of blood without anticoagulants, centrifuging it at 2700-3000 rpm for 10-12 minutes, and using the resulting PRF for surgical closure with silk sutures (30). Khan et al. also used a method involving the extraction of 5 mL of blood, followed by centrifugation at 2700 rpm for 12.5 minutes to obtain PRF, according to the Choukroun method (31). It is important to mention that in all the reviewed studies, implant placement was carried out after tooth extraction and alveolar preservation, except in the trials by Natto et al., Afifi et al., and Eeckhout et al., where it was not clearly specified whether implant placement was subsequently performed (17 , 21 , 30). It is also worth noting that in the studies by Alasqah et al. and Kim et al., no such intervention was performed, as they dealt with third molar extractions, for which implantation is not a standard procedure (26 , 27). Additionally, several studies included supplementary protocols such as the application of chlorhexidine for oral hygiene, in varying concentrations and durations, as well as antibiotic prophylaxis regimens and postoperative management with analgesics and antibiotics. These details were reported in studies by Anderud et al., Gabay et al., Iorio-Siciliano et al., Meloni et al., Natto et al., Maiorana et al., Lahham et al., Areewong et al., Eeckhout et al., Afifi et al., Khan et al., and Kim et al. (11 , 17 , 21 - 23 , 25 , 26 , 29 - 33). In terms of evaluated outcomes, the studies mainly focused on alveolar bone loss, with detailed analysis of horizontal and vertical changes, as well as soft tissue healing. Histological and histomorphometric studies were conducted in some trials to assess new bone formation, the presence of residual graft material, and the inflammatory response (11 , 29). Additionally, implant placement success rate and primary stability were also key outcomes in some studies, while others further explored patient-reported outcomes, such as postoperative pain and discomfort (22 , 27) (Tables 2,3).

Table 2

Table 3

## Discussion

Post-extraction alveolar ridge resorption remains a well-documented and clinically relevant phenomenon that may compromise future implant placement and aesthetic outcomes. Despite advances in extraction techniques and regenerative approaches, dimensional changes of the alveolar ridge-particularly during the first months after extraction-are largely unavoidable. Consequently, alveolar ridge preservation (ARP) has become an essential component of contemporary implant dentistry, especially in cases where immediate implant placement is not feasible or predictable (11 , 13). This systematic review evaluated randomized controlled trials assessing the effectiveness of collagen-based materials and platelet-rich fibrin (PRF) for alveolar ridge preservation compared with spontaneous healing. The results indicate that both biomaterials provide clinical benefits; however, their effectiveness differs depending on the primary treatment objective, extraction site, and whether they are used alone or in combination with additional grafting materials. - Changes in alveolar ridge dimensions Overall, collagen-based approaches-particularly when combined with bone grafts such as deproteinized bovine bone mineral (DBBM)-demonstrated more consistent preservation of alveolar ridge dimensions. Several studies reported reduced vertical and horizontal bone loss compared with spontaneous healing, especially in sites with thin buccal bone walls, where resorption is known to be more pronounced (29 , 33). These findings suggest that collagen membranes or matrices may enhance the stability of grafted sites by acting as a protective barrier, limiting soft tissue ingrowth and supporting guided bone healing. In contrast, the use of collagen alone, without an underlying graft material, showed limited effectiveness in preventing dimensional bone loss. Studies evaluating collagen sponges or matrices as the sole intervention frequently reported no statistically significant differences compared with natural healing (17 , 25). This reinforces the concept that collagen alone lacks sufficient structural support to counteract post-extraction remodeling and should preferably be combined with a bone substitute when dimensional stability is a priority. PRF-based protocols yielded more heterogeneous results. While some studies demonstrated a reduction in horizontal and vertical bone loss when PRF was combined with bone grafts or when advanced or recurrent applications were used (22 , 30 , 31), conventional PRF applied alone did not consistently result in significant dimensional preservation (23 , 27). These findings highlight the importance of application protocol and biomaterial synergy, suggesting that PRF may enhance healing when used as an adjunct rather than as a standalone solution for ridge preservation. - Soft tissue healing and postoperative pain In contrast to its variable effect on hard tissue preservation, PRF showed more consistent benefits in soft tissue healing and postoperative pain reduction. Several studies reported lower pain scores and improved early healing in PRF-treated sites compared with spontaneous healing, particularly during the first postoperative days (22 , 24 , 27 , 30). These effects are likely related to the sustained release of growth factors and cytokines that modulate inflammation, angiogenesis, and tissue regeneration. Collagen-based materials also demonstrated favorable effects on postoperative comfort and soft tissue healing, although these benefits were generally less pronounced than those observed with PRF (26). Nevertheless, collagen matrices may contribute to wound stabilization and epithelialization, especially when used as socket sealers. - Influence of the extraction site The extraction site appears to play a critical role in determining the effectiveness of ridge preservation strategies. The anterior region, characterized by thinner buccal bone walls, is particularly susceptible to resorption and presents higher aesthetic demands. Several studies emphasized that preservation techniques are especially relevant in these areas to maintain gingival contour and support future implant placement (11 , 25 , 33). In this context, PRF may be particularly advantageous in aesthetic zones due to its positive impact on soft tissue healing and postoperative comfort. Conversely, collagen combined with bone grafts appears more suitable for posterior regions, where structural bone stability and volume maintenance are the primary objectives (17 , 29 , 31). - Histological and histomorphometric considerations Only a limited number of studies included histological or histomorphometric evaluations, which restricts definitive conclusions regarding the quality of newly formed bone. Available evidence suggests that grafted sites using DBBM combined with collagen demonstrate greater dimensional stability but may exhibit residual graft particles over time (11 , 29). In contrast, growth factor-based approaches such as PRGF showed higher proportions of newly formed bone, although results were not consistent across all PRF-derived protocols (23 , 24). These findings underline the need for standardized histological assessment methods in future trials. - Comparative effectiveness of collagen and PRF Taken together, the findings of this review indicate that collagen and PRF serve different but complementary roles in alveolar ridge preservation. Collagen-based approaches, particularly when combined with bone grafts, provide greater predictability in preserving alveolar ridge dimensions. PRF, while less consistent in maintaining bone volume, offers clear advantages in soft tissue healing and postoperative pain reduction (13). Therefore, biomaterial selection should be guided by clinical priorities. When dimensional bone stability is critical-such as in posterior regions or sites requiring future implant placement-collagen combined with a bone graft appears to be the preferred option. Conversely, in aesthetic areas where soft tissue healing and patient comfort are paramount, PRF represents a valuable adjunctive therapy. Although five studies were classified as having low risk of bias, the majority of the included trials presented some concerns, particularly regarding deviations from intended interventions and reporting transparency. In addition, most studies had relatively short follow-up periods (2-6 months), which may not fully capture long-term dimensional remodeling. Therefore, the overall certainty of the evidence can be considered moderate to low. Consequently, the conclusions of this review should be interpreted with caution, and clinical decision-making should consider patient-specific factors and site characteristics. - Limitations The main limitation of this systematic review is the heterogeneity among the included studies in terms of biomaterial protocols, measurement methods, and follow-up duration, which limited direct comparison of outcomes. Variability in PRF preparation and collagen application, as well as the frequent combination of these materials with different grafting approaches, made it difficult to isolate their individual effects. In addition, several studies included small sample sizes and short follow-up periods, and some presented methodological concerns related to risk of bias. These factors should be considered when interpreting the results and highlight the need for more standardized long-term randomized controlled trials. Furthermore, the absence of standardized measurement protocols and the frequent combination of biomaterials (for example, PRF or collagen used adjunctively with bone grafts) limited the ability to isolate the independent effect of each material. The variability in reporting outcomes in millimeters versus percentages also reduced comparability across trials. Future studies should adopt standardized dimensional assessment methods and clearly defined intervention protocols to enhance reproducibility and allow quantitative synthesis

## Conclusions

Based on the available evidence, both collagen-based materials and platelet-rich fibrin (PRF) offer clinical advantages over spontaneous healing for alveolar ridge preservation following tooth extraction. Collagen, especially when used in combination with bone grafts such as deproteinized bovine bone mineral, showed a more consistent capacity to limit horizontal and vertical bone resorption, supporting its use when dimensional stability is the primary objective. PRF, on the other hand, demonstrated more variable results in terms of hard tissue preservation, but consistently showed beneficial effects on soft tissue healing and postoperative pain reduction. These properties make PRF particularly useful in aesthetic areas, where rapid soft tissue recovery and patient comfort are of greater relevance. Nevertheless, the heterogeneity among the included studies-regarding biomaterial preparation, application protocols, follow-up duration, and outcome assessment-limits direct comparisons and the strength of the conclusions. Further well-designed randomized controlled trials with standardized biomaterial protocols, longer follow-up periods, and harmonized outcome measures are required to strengthen the certainty of the evidence and enable future meta-analytic comparisons.

## Figures and Tables

**Table 1:** Table Eligibility criteria.

Inclusion criteria	Exclusion criteria
Full text available in English	In vitro studies
Article published in 2014 and onwards	Case report studies
Randomized Controlled Trial (RCT) performed on human adults	Case series studies
Study based on the parameters width of the alveolar ridge and height of the alveolar ridge	Case-control studies
Measurements taken within a defined time frame	Cross-sectional studies
	Non-randomized clinical trials
	RCT performed on animals
	RCT performed on children

1

**Table 2:** Table Characteristics of the included studies.

Author (Year)	Design / Sample	Intervention vs Control	Follow-up	Primary outcomes	Key findings
Anderud et al. (2021)	RCT, n=23	Collagen sponge vs empty socket	12 weeks	Bone and soft tissue loss	No significant benefit of collagen sponge
Gabay et al. (2020)	RCT, n=30	DBBM-C + CMXs vs spontaneous healing	6 months	Ridge width/height, bone formation	Slight reduction of ridge loss, not significant
Iorio-Siciliano et al. (2017)	RCT, n=20	Bio-Oss + membrane vs spontaneous healing	6 months	Ridge width and height	Preservation recommended in thin buccal walls
Meloni et al. (2015)	RCT, n=30	CT graft vs collagen matrix	1 year	Bone dimensions, implant success	No differences; collagen matrix simplified treatment
Llanos et al. (2019)	RCT, n=65	DBBM vs DBBM-C	4 months	Horizontal ridge width	DBBM non-inferior to DBBM-C
Maiorana et al. (2017)	Multicenter trial, n=7	DBBM + collagen membrane	6 months	Ridge dimensions, histology	Stable ridge with good healing
Eeckhout et al. (2024)	RCT, n=18	Collagen matrix vs gelatin sponge	4 months	Soft tissue and bone loss	Both effective; collagen better for soft tissue
Kim et al. (2020)	RCT, n=31	Collagen sponge vs no sponge	14 weeks	Pain, healing, bone density	Reduced pain and probing depth
Natto et al. (2017)	RCT, n=28	CMS+FDBA vs CS+FDBA	4 months	Ridge width, soft tissue thickness	Both reduced ridge resorption
Alasqah et al. (2024)	RCT, n=60	PRF vs PRF+collagen vs control	3 months	Ridge height/width, pain	No bone differences; pain reduced
Lahham et al. (2023)	RCT, n=20	A-PRF+ vs A-PRF+ + C-PRF	3 months	Ridge alterations	Recurrent PRF reduced ridge changes
Abad et al. (2023)	RCT, n=27	L-PRF vs spontaneous healing	4 months	Ridge width and height	No benefit of L-PRF
Areewong et al. (2019)	RCT, n=36	PRF vs normal healing	2 months	New bone formation	No significant difference
Anitua et al. (2015)	RCT, n=60	PRGF vs blood clot	10-12 weeks	Bone regeneration, pain	PRGF improved regeneration and healing
Afifi et al. (2025)	RCT, n=26	PRF vs FGG	6 months	Dimensional changes, pain	PRF better buccolingual preservation
Khan et al. (2024)	RCT, n=100	DFDBA + PRF vs natural healing	6 months	Bone density and resorption	DFDBA + PRF superior

DBBM-C: Deproteinized bovine bone mineral with 10% collagen; DBBM: Demineralized bovine bone mineral; CMXs: Procaine collagen membrane; Bio-Oss, Geistlich: Bovine xenograft with 10% collagen; Bio-Gide, Geistlich: Collagen membrane; CMS: Collagen matrix; CM: Collagen matrix; CS: Collagen sponge; ARP: Alveolar ridge preservation; FDBA: Freeze-dried bone allograft; PRF: Platelet-rich fibrin; A-PRF+: Advanced platelet-rich fibrin; C-PRF: Concentrated platelet-rich fibrin with recurrent applications; PROM’S: Patient-reported outcome measures; L-PRF: Leukocyte- and platelet-rich fibrin; FGG: Free gingival grafts; DFDBA: Demineralized freeze-dried bone allograft.

**Table 3:** Table Comparative Table of Vertical and Horizontal Bone Loss According to the Material Used in Each Study.

Author (Year)	Material / Technique	Horizontal bone loss	Vertical bone loss	Statistical significance
Anderud et al. (2021)	Collagen sponge vs no graft	1.15 mm vs 0.57 mm	1.74 mm vs 1.90 mm	Not significant (P > 0.05)
Gabay et al. (2020)	DBBM-C + collagen membrane vs spontaneous healing	1.19-1.61 mm vs 1.96-2.27 mm	0.14 mm vs 0.98 mm	Not significant
Iorio-Siciliano et al. (2017)	Bio-Oss + membrane vs spontaneous healing	Significant reduction when buccal wall ≤1 mm	Significant reduction when buccal wall ≤1 mm	Significant (P < 0.05)
Meloni et al. (2015)	CT graft vs collagen matrix	1.60 mm vs 1.47 mm	No relevant differences	Not significant
Anitua et al. (2015)	PRGF vs spontaneous healing	96.5% vs 74.6% bone regeneration	63.1% vs 35.6%	Significant (P < 0.001)
Alasqah et al. (2024)	PRF vs PRF+collagen vs control	No meaningful changes	No meaningful changes	Not significant
Natto et al. (2017)	CMS + FDBA vs CS + FDBA	Slightly lower loss with CMS	Slightly lower loss with CMS	Not significant
Afifi et al. (2025)	PRF vs free gingival graft	0.8 mm vs 1.6 mm	0.3 mm vs 1.4 mm	Horizontal significant; vertical NS
Khan et al. (2024)	DFDBA + PRF vs spontaneous healing	11.9 mm vs 14.0 mm	12.2 mm vs 14.6 mm	Significant (P < 0.05)
Llanos et al. (2019)	DBBM-C vs DBBM	Similar values at all levels	Similar values	Not significant
Maiorana et al. (2017)	DBBM + collagen matrix vs spontaneous healing	1.21 mm vs 3.79 mm	0.46 mm vs 1.24 mm	Significant (P < 0.01)
Abad et al. (2023)	L-PRF vs spontaneous healing	No relevant differences	No relevant differences	Not significant
Areewong et al. (2019)	PRF vs spontaneous healing	New bone: 31.3% vs 26.3%	—	Not significant
Kim et al. (2020)	Collagen sponge vs no sponge	5.55 mm vs 7.13 mm	Not reported	Horizontal significant
Eeckhout et al. (2024)	Collagen matrix vs gelatin sponge	Similar loss at all levels	Not reported	Not significant
Lahham et al. (2023)	A-PRF+ vs A-PRF+ + C-PRF	1.9 mm vs 2.9 mm	1.0 mm vs 1.8 mm	Significant (P < 0.05)

NR: Not reported; % values correspond to histological bone formation.

## Data Availability

The data presented in this study are available from the corresponding author upon reasonable request.
